# Effect of adding hydrochlorothiazide to usual treatment of patients with acute decompensated heart failure: a randomized clinical trial

**DOI:** 10.1038/s41598-021-96002-6

**Published:** 2021-08-13

**Authors:** Diogo Silva Piardi, Maurício Butzke, Ana Carolina Martins Mazzuca, Bruna Sessim Gomes, Sofia Giusti Alves, Bruno Jaskulski Kotzian, Eduarda Chiesa Ghisleni, Vanessa Giaretta, Priscila Bellaver, Gabrielle Aguiar Varaschin, Arthur Pereira Garbin, Luís Beck-da-Silva

**Affiliations:** 1 Universidade Federal do Rio Grande do Sul, Porto Alegre, Brazil; 2 Serviço de Cardiologia, Hospital de Clínicas de Porto Alegre (HCPA), Rua Ramiro Barcelos, 2350, Porto Alegre, 90035-003 Brazil

**Keywords:** Cardiology, Nephrology

## Abstract

Acute decompensated heart failure (ADHF) is the leading cause of hospitalization in patients aged 65 years or older, and most of them present with congestion. The use of hydrochlorothiazide (HCTZ) may increase the response to loop diuretics. To evaluate the effect of adding HCTZ to furosemide on congestion and symptoms in patients with ADHF. This randomized clinical trial compared HCTZ 50 mg versus placebo for 3 days in patients with ADHF and signs of congestion. The primary outcome of the study was daily weight reduction. Secondary outcomes were change in creatinine, need for vasoactive drugs, change in natriuretic peptides, congestion score, dyspnea, thirst, and length of stay. Fifty-one patients were randomized—26 to the HCTZ group and 25 to the placebo group. There was an increment of 0.73 kg/day towards additional weight reduction in the HCTZ group (HCTZ: − 1.78 ± 1.08 kg/day vs placebo: − 1.05 ± 1.51 kg/day; *p* = 0.062). In post hoc analysis, the HCTZ group demonstrated significant weight reduction for every 40 mg of intravenous furosemide (HCTZ: − 0.74 ± 0.47 kg/40 mg vs placebo: − 0.33 ± 0.80 kg/40 mg; *p* = 0.032). There was a trend to increase in creatinine in the HCTZ group (HCTZ: 0.50 ± 0.37 vs placebo: 0.27 ± 0.40; *p* = 0.05) but no significant difference in onset of acute renal failure (HCTZ: 58% vs placebo: 41%; *p* = 0.38). No differences were found in the remaining outcomes. Adding hydrochlorothiazide to usual treatment of patients with acute decompensated heart failure did not cause significant difference in daily body weight reduction compared to placebo. In analysis adjusted to the dose of intravenous furosemide, adding HCTZ 50 mg to furosemide resulted in a significant synergistic effect on weight loss.

Trial registration: The Brazilian Clinical Trials Registry (ReBEC), a publically accessible primary register that participates in the World Health Organization International Clinical Trial Registry Platform; number RBR-5qkn8h. Registered in 23/07/2019 (retrospectively), http://www.ensaiosclinicos.gov.br/rg/RBR-5qkn8h/.

## Introduction

Acute decompensated heart failure (ADHF) is the leading cause of hospitalization in patients aged 65 years or older^1^, accounting for approximately 800,000 admissions in the United States, with a substantial impact on both morbidity and mortality^2^. According to estimates, 1 every 2 patients is readmitted within 6 months with in-hospital mortality ranging from 4 to 12%^3^. In Brazil, mortality rates are even higher^4,5^. Following hospital discharge, 35% of patients with ADHF are estimated to die within 1 year^3^.

ADHF may have different presentations, and patients most commonly present with congestion^6^. Given its association with poor renal function, congestion may have therapeutic and prognostic implications, especially when sustained^7^. This is possibly due to increased systemic and portal venous pressure, ascites, and increased intra-abdominal pressure, leading to cardiorenal syndrome^8,9^.

Loop diuretics act on the sodium–potassium–chloride (Na–K–2Cl) cotransporter in the thick ascending limb of the loop of Henle and produce diuresis by blocking sodium reabsorption^10^. Furosemide, a loop diuretic, is often called a high-ceiling diuretic because it is more effective than other diuretics. It plays the leading role in the treatment of congestion, being used in more than 90% of patients hospitalized with heart failure^11^. In addition to their diuretic effect, loop diuretics produce vasodilation on smooth muscle cells in the vessels. There is also an effect on the activation of the renin–angiotensin–aldosterone system (RAAS), which has a positive side, i.e., adjustment of the glomerular flow in the nephron, and a negative side, i.e., neurohumoral activation leading to the perpetuation of deleterious effects on the patient with heart failure^12^.

However, long-term use of diuretics in patients with ADHF is quite common, generating potential mechanisms of diuretic resistance, in which the diuretics do not achieve the desired effect, manifested by low urine sodium concentrations despite the recommended maximum doses. Several mechanisms have been proposed to explain such process, including reduced diuretic efficacy, salt retention, rebound effect, and nephron remodeling^12,13^. Also, high doses may be associated with adverse outcomes^14^. An alternative approach used in clinical practice is sequential nephron blockade, which consists of the combination of diuretic classes, such as loop and thiazide diuretics, acting on the distal convoluted tubule, and aldosterone antagonists, acting on the collecting duct^11^.

In the DOSE (Diuretic Strategies in Patients with Acute Decompensated Heart Failure) trial^14^, there was an absolute increase in the use of thiazide diuretics by 11%, as well as a trend towards a greater use of thiazide diuretics in patients on low-dose loop diuretics. However, less than 400 patients have been included to date in studies, mostly observational, assessing the combination of diuretics in patients with ADHF^15^. Thus, the present study aimed to evaluate the effect of hydrochlorothiazide (HCTZ) added to furosemide versus furosemide alone on congestion, measured by weight reduction, in patients with ADHF.

## Methods

### Study design

A randomized, single-center, parallel, double-blind, placebo-controlled clinical trial was conducted.

### Participants

Patients admitted to the emergency department at Hospital de Clínicas de Porto Alegre, southern Brazil, with ADHF and systolic dysfunction (ejection fraction [EF] ≤ 45%) were included if they met the following criteria: age ≥ 18 years, diagnosis of ADHF, emergency department admission < 18 h, endogenous creatinine clearance > 30 mL/min, serum potassium ranging from 3.5 to 5.1 mEq/L, and signs of congestion. Congestion was based on the presence of at least two of the following: lower limb edema; crackles on lung auscultation or chest radiogram with signs of congestion; jugular vein distention or hepatojugular reflux; weight gain > 4.5 kg compared to usual weight; orthopnea and/or paroxysmal nocturnal dyspnea.

Patients were excluded if they had serum sodium > 145 mEq/L, Bartter or Gitelman syndrome, type IV renal tubular acidosis, history of sulfa allergy, current acute coronary syndrome, previous history of gout, pregnancy or lactation, associated septic shock, liver dysfunction (defined as elevated transaminases three times above the reference value), or inability for enteral intake. The study was approved by the Hospital de Clínicas de Porto Alegre Research Ethics Committee (number 45773815.7.0000.5327) and was performed in accordance with Good Clinical Practice guidelines and following the principles of Declaration of Helsinki for protection of research subjects.

### Intervention and logistics

**Figure 1:**
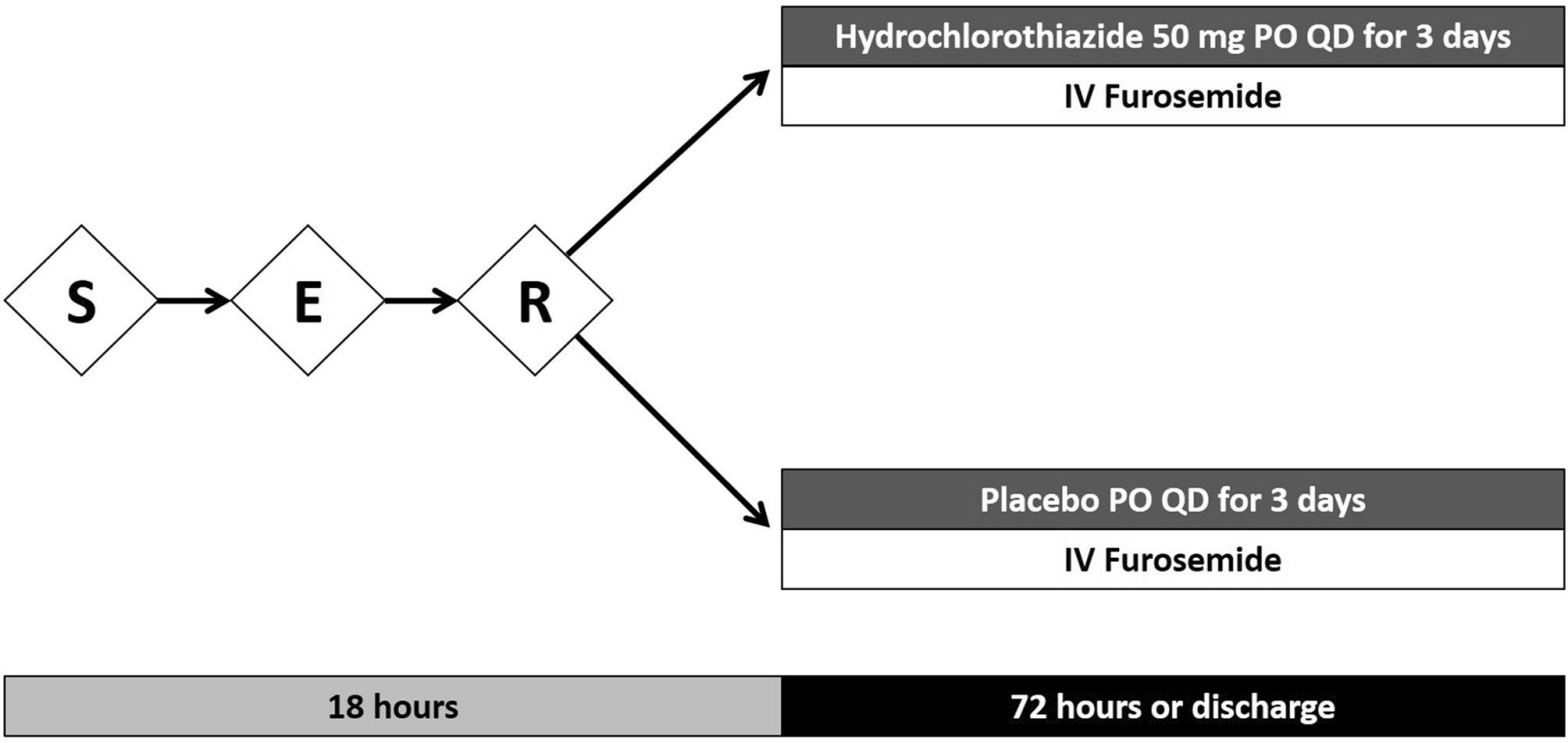
Logistics and interventions. *S* screening, *E* enrollment, *R* randomization, *PO* oral administration, *QD* once daily.

Patients were invited to participate if they met the inclusion criteria for the study and had no exclusion criteria. After signing a written informed consent by the patient or a legally authorized representative, the enrolled patients were randomly allocated to two groups: HCTZ 50 mg or placebo (both oral). Dose of intravenous furosemide and additional therapies were at the discretion of the patient's medical team. The duration of the intervention was 3 days or until hospital discharge if occurring before 3 days of admission (Fig. 1).

An external investigator using a web-based software generated randomized sequences, divided in two blocks, and the resulting spreadsheet was sent to the pharmacist responsible for medications—HCTZ 50 mg and placebo, both stored inside identical numbered vials—and who had no scientific involvement in the study (Spengler Pharmacy, Porto Alegre, Brazil). All investigators and patients were blinded to the treatment assignment.

### Follow-up

**Figure 2:**
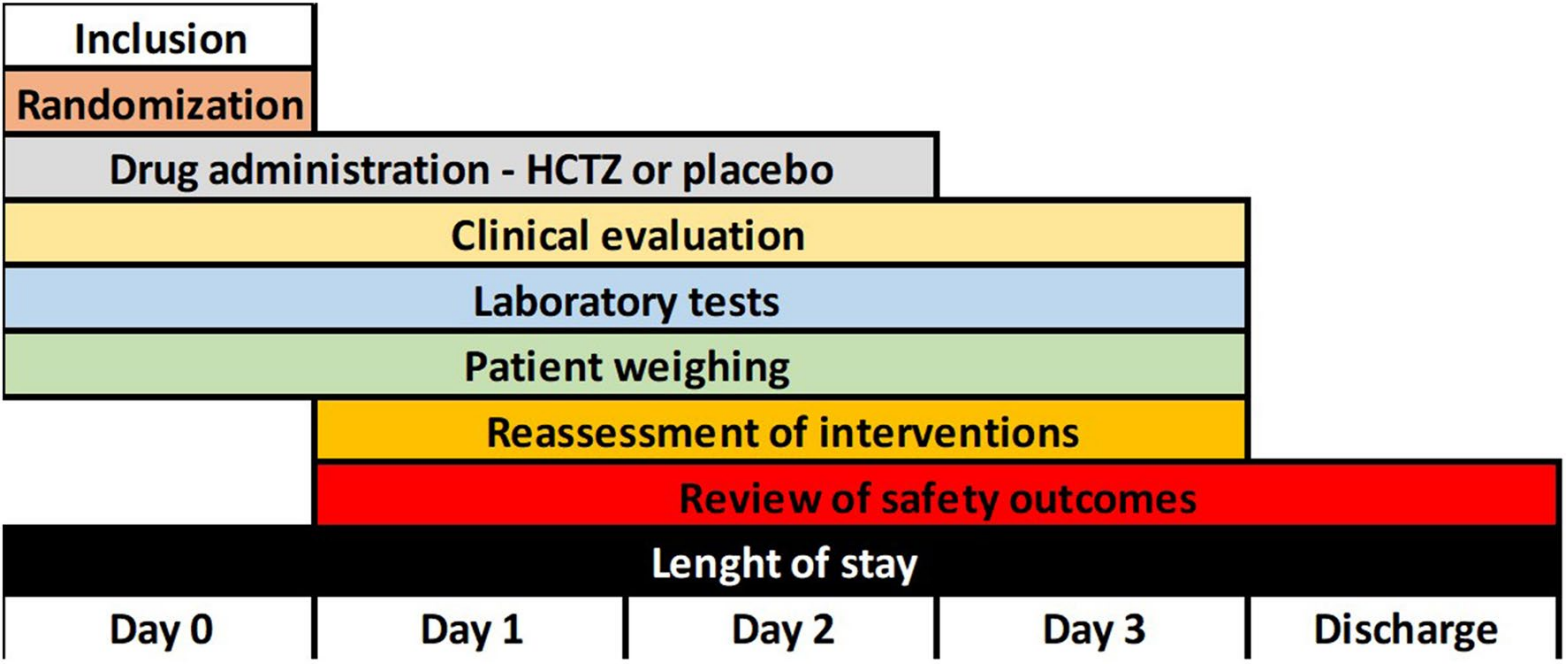
Study flow diagram.

Patients were monitored as follows (Fig. 2):

- Day 0: patient inclusion, randomization, drug administration, assessment of clinical variables, patient weighing, and baseline laboratory tests.
- Days 1 and 2: patient reassessment, order for laboratory tests, review of safety outcomes, drug administration, patient weighing, and reassessment of interventions.
- Day 3: patient reassessment, order for laboratory tests, review of safety outcomes, drug administration, patient weighing, and reassessment of interventions.
- Discharge: review of safety outcomes, length of stay.

### Outcomes

The primary outcome of the study was daily weight change for 3 days, measured daily on the same scale (Mallory, Maranguape, Brazil). The secondary outcomes were change in creatinine, need for vasoactive drugs (sodium nitroprusside, nitroglycerin, dobutamine, milrinone, vasoactive amines), change in natriuretic peptides, congestion score^16^, dyspnea scale (Likert-type), thirst scale, and length of stay. Finally, safety outcomes were in-hospital mortality, hypernatremia (sodium > 145 mEq/L), hypokalemia (potassium < 3.5 mEq/L), hyperkalemia (potassium > 5.5 mEq/L), increase in creatinine > 0.3 mg/dL was considered as worsening renal function, the need for hemodialysis as acute renal failure, and ventricular arrhythmias.

### Sample size calculation

Sample size was calculated using WinPepi, version 11.1 (www.brixtonhealth.com/pepi4windows.html). Based on a study conducted by Dormans et al.^17^, the statistical parameters used were an expected weight loss difference between groups of 0.5 kg per day for a total of 1.5 kg within 3 days, a power of 80% and a significance level of 0.05, standard deviations of 0.3 kg in the control group and 0.8 kg in the intervention group, and possible loss to follow-up. Thus, the minimum sample required was 50 patients.

### Statistical analysis

Data were reported as mean and standard deviation, except for asymmetric variables, which were described as median and interquartile range. Student's t-test and analysis of variance (ANOVA) were used for parametric variables, as appropriate. Mann–Whitney U and Kruskal–Wallis tests were used for nonparametric variables. For categorical variables, chi-square and Fisher's exact tests were used as appropriate. All analyses were based on intention-to-treat principles. Analyses were performed using SPSS, version 18.0 (SPSS Inc., Chicago, USA).

## Results

**Figure 3:**
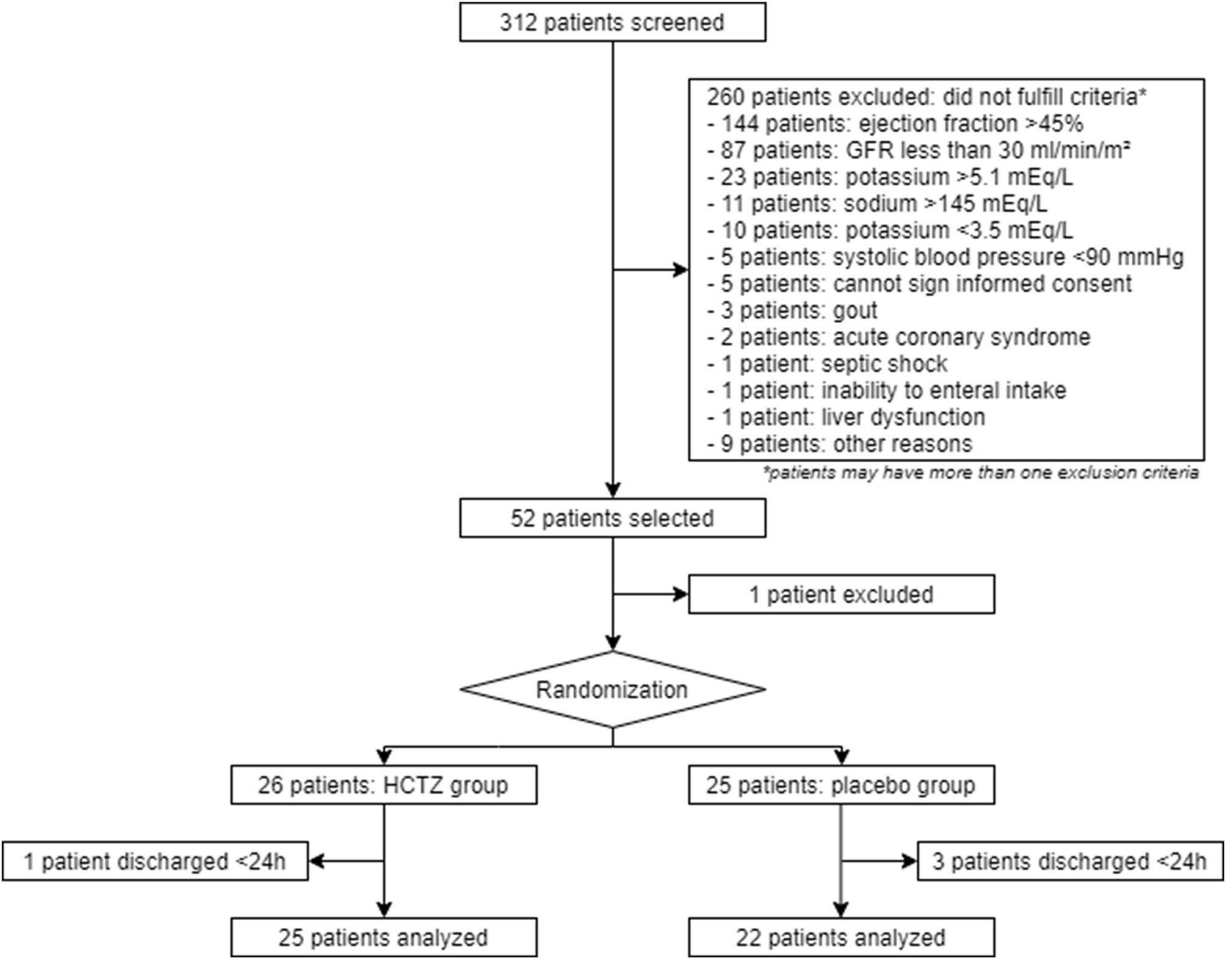
Study flow diagram.

In this study, 312 patients were screened from September 2015 to December 2019, and 52 patients met the inclusion criteria, reaching the required sample size. One patient was excluded before randomization because of gastrointestinal bleeding. Fifty-one patients were randomized—26 to the intervention group and 25 to the control group (Fig. 3).

Patients were mostly white, and their mean age was 64 years. Most of them had hypertension, moderate ventricular dysfunction, history of smoking, and preserved renal function (Tables 1 and 2).

**Table 1:** Demographic and clinical characteristics.

Characteristic	HCTZ (n = 26)	Placebo (n = 25)	*p*-value
Age (years)	64 ± 16	64 ± 10	0.94
Male gender (%)	69	48	0.12
White race (%)	92	76	0.14
Baseline NYHA	2.7 ± 0.8	2.3 ± 0.9	0.11
Ischemic etiology (%)	50	44	0.67
Hypertension (%)	73	72	0.93
Diabetes mellitus (%)	27	44	0.20
Ejection fraction (%)	30 ± 8	31 ± 10	0.50
ICD (%)	7.7	12	0.67
CRT (%)	0	4	0.49
Atrial fibrillation (%)	39	44	0.69
Previous stroke (%)	15	20	0.73
Current/past smoking (%)	65	60	0.69
Beta-blocker (%)	85	80	0.73
ACEI/ARB (%)	92	76	0.14
Outpatient furosemide (mg)	58 ± 57	75 ± 73	0.50
Outpatient spironolactone (mg)	8 ± 12	14 ± 15	0.15
Weight (kg)	81 ± 25	78 ± 19	0.65
Systolic blood pressure (mm Hg)	117 ± 17	122 ± 22	0.32
Diastolic blood pressure (mm Hg)	72 ± 14	73 ± 13	0.91
Heart rate (bpm)	85 ± 19	91 ± 21	0.31
Sodium (mEq/L)	140 ± 4	139 ± 3	0.41
Potassium (mEq/L)	4.5 ± 0.5	4.6 ± 0.5	0.43
Urea (mg/dL)	50 ± 23	54 ± 18	0.55
Creatinine (mg/dL, mean ± SD)	1.23 ± 0.29	1.24 ± 0.34	0.94

Plus–minus values are means ± standard deviation.

*ACEI* angiotensin-converting enzyme inhibitor, *ARB* angiotensin II receptor blocker, *CRT* cardiac resynchronization therapy, *ICD* implantable cardioverter-defibrillator, *NYHA* New York Heart Association classification.

**Table 2:** Therapies during the study.

Medication	HCTZ (n = 26)	Placebo (n = 25)	*p*-value
Endovenous furosemide (3-day total dose, mg)	259 ± 143	246 ± 141	0.62
Endovenous furosemide (3-day total dose/kg, mg)	3.25 ± 1.47	3.25 ± 1.83	*p* = 0.997
Spironolactone (3-day total dose, mg)	26 ± 31	28 ± 37	0.94
Norepinephrine (%)	3.8	0	1.00
Sodium nitroprusside (%)	7.7	4.0	1.00
Milrinone (%)	3.8	0	1.00
Endovenous nitroglycerin (%)	7.7	8.0	1.00

Plus–minus values are means ± standard deviation.

**Figure 4:**
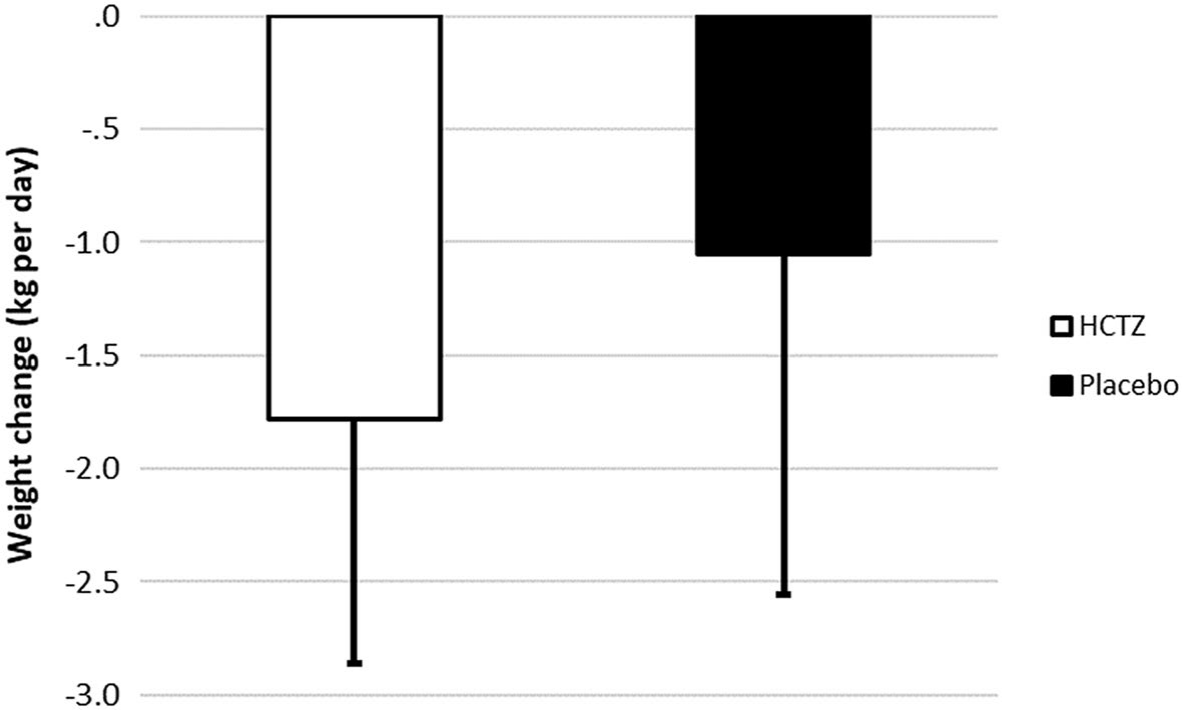
Weight change per day in the HCTZ and placebo groups. Values are reported as mean ± standard deviation (kg/day). HCTZ: − 1.78 ± 1.08 kg/day; placebo: − 1.05 ± 1.51 kg/day; *p* = 0.062.

Regarding the primary outcome, there was a trend towards greater weight reduction in the HCTZ group compared to the placebo group (HCTZ: − 1.78 ± 1.08 vs placebo: − 1.05 ± 1.51 kg/day; *p* = 0.062) (Table 3, Fig. 4).

**Table 3:** Study outcomes.

Outcome	HCTZ	Placebo	*p*-value
**Primary**			
Weight change/day	− 1.78 ± 1.08	− 1.05 ± 1.51	0.062
**Secondary**			
Length of stay (days)	9 ± 8	8 ± 9	0.37
Change in creatinine (mg/dL)	0.50 ± 0.37	0.27 ± 0.40	0.05
Need for vasoactive drugs (%)	19.2	12.0	0.70
Congestion score	− 5.4 ± 4.6	− 4.8 ± 4.6	0.68
Change in dyspnea scale	− 4.7 ± 2.7	− 3.2 ± 3.6	0.14
Thirst scale	− 1.7 ± 4.5	0.5 ± 3.8	0.21
Change in natriuretic peptides (%)^a^	− 11.1 ± 100.3	− 33.3 ± 50.9	0.83
**Safety**			
In-hospital mortality (%, [n])	3.8 (1)	0 (0)	1.00
Hypernatremia (%)	0	4.8	0.47
Hypokalemia (%)	3.8	4.5	1
Hyperkalemia (%)	0	0	1
Increase in creatinine > 0.3 mg/dL (%)	58	41	0.38
Ventricular arrhythmias (%)	3.8	4	1.00
Hemodialysis (%)	3.8	0	1.00

Plus–minus values are means ± standard deviation.

^a^Natriuretic peptides were collected in all patients at baseline and 55% of study populations had a 3-day or discharge follow-up sample collected, with no difference between groups.

**Figure 5:**
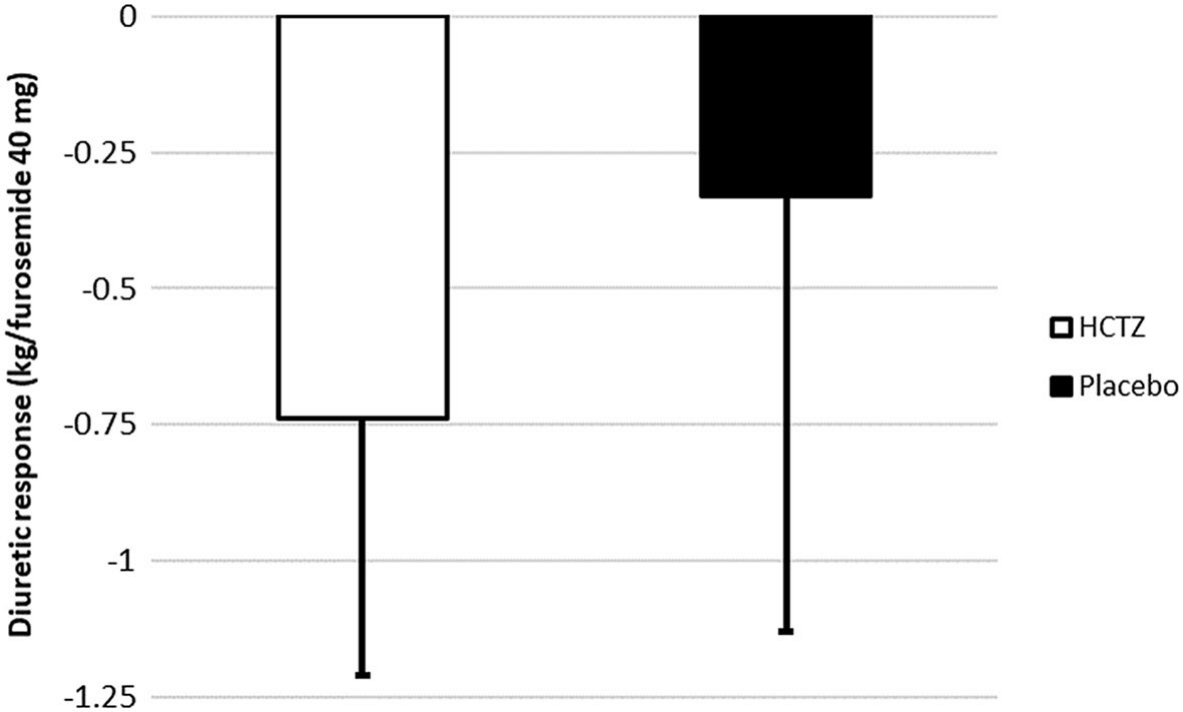
Diuretic response—weight change for every 40 mg of furosemide in the HCTZ and placebo groups. Values are reported as mean ± standard deviation (kg/40 mg of furosemide). HCTZ: − 0.74 ± 0.47 kg/40 mg; placebo: − 0.33 ± 0.80 kg/40 mg; *p* = 0.032.

For the ratio between total weight loss and total prescribed dose of intravenous furosemide, a post hoc analysis showed a statistically significant additional weight reduction for every 40 mg of intravenous furosemide (HCTZ: − 0.74 ± 0.47 kg/40 mg vs placebo: 0.33 ± 0.80 kg/40 mg; *p* = 0.032) (Fig. 5).

There was no difference in length of stay, in-hospital mortality, congestion score, electrolyte disturbances, need for hemodialysis, ventricular arrhythmias, need for vasoactive drugs, and change in natriuretic peptides. Likewise, no difference was found in dyspnea or thirst between the groups. In absolute values, there was an increase in creatinine with borderline significance (HCTZ: 0.50 ± 0.37 vs placebo: 0.27 ± 0.40; *p* = 0.05). However, when comparing an increase in creatinine > 0.3 mg/dL, there was no statistically significant difference between the groups (HCTZ: 58% vs placebo: 41%; *p* = 0.38) (Table 3).

## Discussion

**Figure 6:**
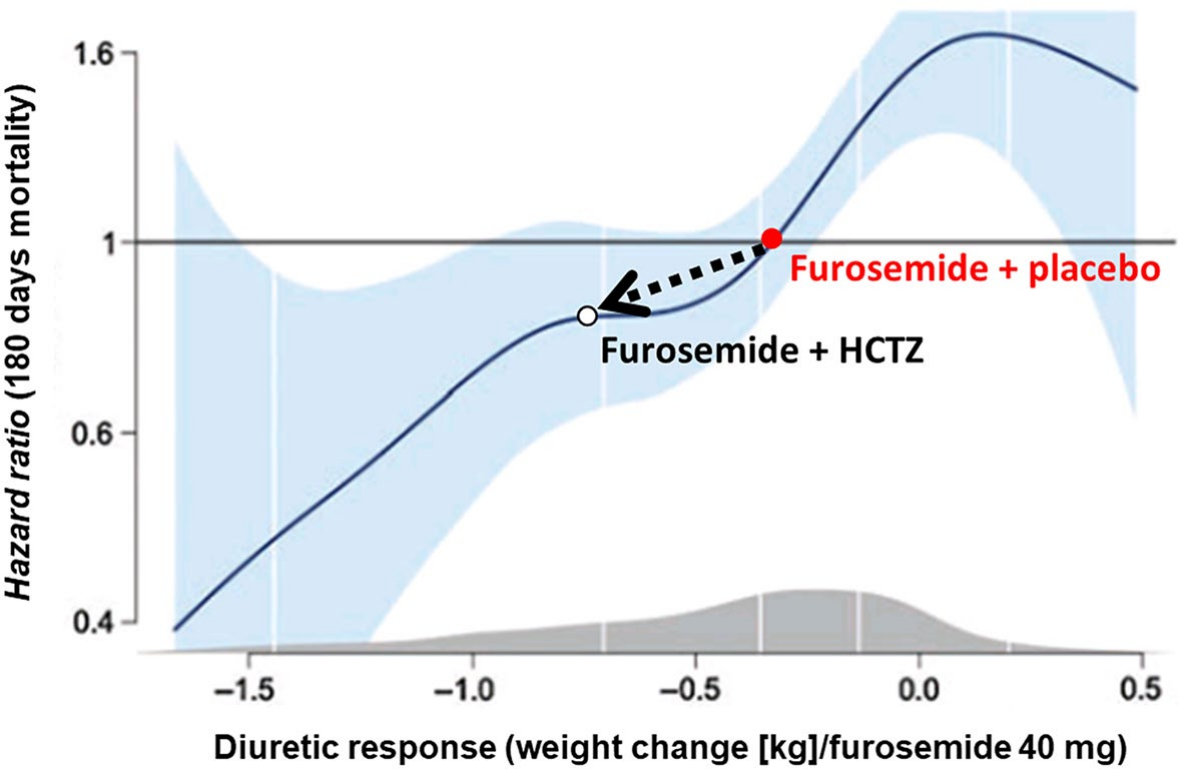
Effect of adding Hydrochlorothiazide (in our trial) plotted over Valente et al. data estimation of mortality based on diuretic response. Adapted from Valente et al.^18^, which suggested lower mortality with greater diuretic response.

This randomized, placebo-controlled clinical trial evaluated the effect of adding HCTZ to usual treatment in patients with ADHF. We have demonstrated that the mean daily weight loss, for the first 3 days of admission, were 0.7 kg/day higher than in placebo group. For each 40 mg of intravenous furosemide used in the treatment of ADHF, HCTZ provided a statistically significant additional effect of 0.4 kg/day. Diuretic response at the beginning of ADHF therapy has been associated, with less symptoms, better quality of life^14^, less signs of congestion^16^, less admissions^7^, and with reduced mortality^18^.Our study has found a better diuretic response on the first days of therapy by adding HCTZ 50 mg per day to the usual care of patients with ADHF. When our data, based on the effect of adding HCTZ on weight change for each 40 mg of furosemide, is compared to data of a larger study investigating prognosis of diuretic response, regardless of HCTZ use or not, we have found that our data perfectly match with Valente’s data^18^. We, therefore, could possibly raise the hypothesis that having a better diuretic response at the early phase of therapy, by adding HCTZ, could possibly positively impact on patients’ prognosis (Fig. 6).

Other diuretics have been investigated in the setting of ADHF. Initial data were encouraging for the use of spironolactone in a diuretic dose, suggesting that it could translate into less signs of congestion and lower levels of natriuretic peptides^19^. Moreover, spironolactone was found to be a predictor of diuretic response^18^. The ATHENA-HF (Aldosterone Targeted Neurohormonal Combined with Natriuresis Therapy in Heart Failure) trial was then conducted to assess spironolactone 100 mg added to usual treatment of ADHF, and previous findings were not confirmed. There was no reduction in natriuretic peptides nor was there any additional weight loss when compared to placebo^20^.

The use of loop diuretics for the management of ADHF is well established. In the DOSE study, a comparison between high and low diuretic doses showed no differences in the primary outcomes of renal function and symptoms nor in weight loss, which was a secondary outcome in the study. However, a post hoc analysis demonstrated a greater use of thiazide diuretics in patients in the low-dose furosemide group, with a trend towards statistical significance^14^. Such finding might be an explanation for neutrality in that study and adds another element to confirm the findings of our study.

Our study has some limitations. It was a single-center study with a relatively small number of patients. Significance was not reached because the standard deviation was larger than predicted in sample size calculation, based on a previous study^17^. According to the sample-size calculation, we estimated standard deviation (SD) of 0.3 kg in control group and 0.8 kg in intervention group. However, our results have shown SD of 1.08 kg for intervention group and 1.51 kg for placebo group, both being much larger that the assumption. Although the primary outcome reached borderline significance, a post hoc analysis adjusted to the dose of intravenous furosemide showed a statistically significant result, which is consistent with the hypothesis of synergy when adding HCTZ to furosemide, with a clinically relevant effect on daily weight loss.

Regarding safety outcomes, there was a greater change in creatinine levels in patients using HCTZ. However, there was no difference between the groups when analyzing onset of worsening renal failure, defined as an increase in creatinine > 0.3 mg/dL. According to previous studies, increased creatinine is not related to poor prognosis when patients have reduced congestion^18,21^, which occurred more consistently in patients using HCTZ. In terms of quality of life during the process of compensation in ADHF, there was no difference in thirst between the groups. In previous studiesof patients with ADHF, thirst was exacerbated during a non-pharmacologic intervention targeting decongestion^22,23^.

Other congestion-related data did not differ between the groups; however, congestion is difficult to assess. The development of new technologies for assessing congestion will contribute to the definition of outcomes in future studies. Moreover, there was neither quantification of diuresis nor assessment of fractional excretion of sodium, which are difficult to measure in an emergency room setting, where the study was conducted. Our HCTZ study was designed to assess weight reduction, with no power for hard outcomes. However, the result could be interpreted as a promising one for ADHF patients.

## Conclusions

We have demonstrated that, by adding HCTZ to usual treatment of patients with ADHF, the mean daily weight loss, for the first 3 days of admission, were **0.73** kg higher than in placebo group. This result did not reach statistical significance. In analysis adjusted to the dose of intravenous furosemide, adding HCTZ 50 mg to usual treatment resulted in a synergistic effect on weight loss, with a statistically significant increase in the diuretic effect for every 40 mg of intravenous furosemide used in patients with ADHF.

## Abbreviations

ADHF: Acute decompensated heart failure
ACEI: Angiotensin-converting enzyme inhibitor
ARB: Angiotensin II receptor blocker
ATHENA-HF: Aldosterone targeted neurohormonal combined with natriuresis therapy in heart failure trial
CRT: Cardiac resynchronization therapy
DOSE: Diuretic strategies in patients with acute decompensated heart failure trial
HCTZ: Hydrochlorothiazide
ICD: Implantable cardioverter-defibrillator
NYHA: New York Heart Association classification
RAAS: Renin–angiotensin–aldosterone system
