# Author Correction: Effect of adding hydrochlorothiazide to usual treatment of patients with acute decompensated heart failure: a randomized clinical trial

**DOI:** 10.1038/s41598-021-96943-y

**Published:** 2021-08-24

**Authors:** Diogo Silva Piardi, Maurício Butzke, Ana Carolina Martins Mazzuca, Bruna Sessim Gomes, Sofia Giusti Alves, Bruno Jaskulski Kotzian, Eduarda Chiesa Ghisleni, Vanessa Giaretta, Priscila Bellaver, Gabrielle Aguiar Varaschin, Arthur Pereira Garbin, Luís Beck-da-Silva

**Affiliations:** 1 Universidade Federal do Rio Grande do Sul, Porto Alegre, Brazil; 2 Serviço de Cardiologia, Hospital de Clínicas de Porto Alegre (HCPA), Rua Ramiro Barcelos, 2350, Porto Alegre, 90035-003 Brazil

Correction to: *Scientific Reports* 10.1038/s41598-021-96002-6, published online 13 August 2021

The original version of this Article contained an error in the Funding section.

“This study was supported by Fundo de Incentivo a Pesquisa at Hospital de Clínicas de Porto Alegre (FIPE/HCPA).”

now reads:

“This study was partially funded by the *Coordenação de Aperfeiçoamento de Pessoal de Nível Superior* (Coordination for the Improvement of Higher Education Personnel; CAPES) - Brasil - Finance Code 001; partially funded by Hospital de Clínicas de Porto Alegre (Fundo de Incentivo à Pesquisa e Eventos; FIPE/HCPA), No. 2015-0212."

The original Article has been corrected.

